# Diagnosing and monitoring pancreatic cancer through cell-free DNA methylation: progress and prospects

**DOI:** 10.1186/s40364-023-00528-y

**Published:** 2023-10-05

**Authors:** María Victoria García-Ortiz, Pablo Cano-Ramírez, Marta Toledano-Fonseca, Enrique Aranda, Antonio Rodríguez-Ariza

**Affiliations:** 1 Maimónides Biomedical Research Institute of Córdoba (IMIBIC), Córdoba, Spain; 2 Andalusia-Roche Network Mixed Alliance in Precision Medical Oncology, Sevilla, Spain; 3 Cancer Network Biomedical Research Center (CIBERONC), Madrid, Spain; 4 Medical Oncology Department, Reina Sofía University Hospital, Córdoba, Spain; 5 Department of Medicine, Faculty of Medicine, University of Córdoba, Córdoba, Spain

**Keywords:** Biomarkers, Cell-free DNA, Epigenetics, Liquid biopsy, Methylation, Pancreatic cancer

## Abstract

Pancreatic cancer is one of the most challenging cancers due to its high mortality rates. Considering the late diagnosis and the limited survival benefit with current treatment options, it becomes imperative to optimize early detection, prognosis and prediction of treatment response. To address these challenges, significant research efforts have been undertaken in recent years to develop liquid-biopsy-based biomarkers for pancreatic cancer. In particular, an increasing number of studies point to cell-free DNA (cfDNA) methylation analysis as a promising non-invasive approach for the discovery and validation of epigenetic biomarkers with diagnostic or prognostic potential. In this review we provide an update on recent advancements in the field of cfDNA methylation analysis in pancreatic cancer. We discuss the relevance of DNA methylation in the context of pancreatic cancer, recent cfDNA methylation research, its clinical utility, and future directions for integrating cfDNA methylation analysis into routine clinical practice.

## Introduction

Pancreatic cancer is the tumor with the 3rd highest mortality rate in developed countries and the tumor with the lowest 5-year survival (9%) [1, 2]. In Europe, approximately 95,000 lives are lost each year due to this condition [3]. Over the past few years, there has been a rise in pancreatic cancer incidence, and the age of most patients diagnosed with this tumor ranges between 70 and 80 years [4]. *BRCA1* and *BRCA2* mutations are the most common genetic alterations in familial pancreatic cancer, in which there is an inherited susceptibility to the disease and that accounts for approximately 10% of cases [5]. On the other hand, sporadic pancreatic cancer accounts for the majority of cases and occurs without any known inherited genetic predisposition, with chronic pancreatitis, diabetes, tobacco, obesity, *H. pylori* infection, and diet as the most important risk factors [6]. Pancreatic cancer is a difficult tumor to diagnose in the initial stages, very aggressive, fast growing and with a poor prognosis. The majority of patients are typically diagnosed with either locally advanced or metastatic disease, with only a small percentage, around 15–20%, being considered operable at the time of diagnosis. However, results of surgery alone are disappointing, as patients often experience early relapse, resulting in a relatively short median survival of only 15 to 20 months [7]. For those diagnosed with metastatic disease, the median overall survival from the time of diagnosis is even shorter, averaging around 4.6 months [8].

Pancreatic ductal adenocarcinoma (PDAC) is the most common type of pancreatic cancer, accounting for over 80% of all cases [9]. *KRAS* (Kirsten rat sarcoma viral oncogene homolog) mutations play a critical role in PDAC, being present in more than 90% of cases and considered one of the key driving factors in the development of the disease [10]. Hence, the presence of a *KRAS* mutation in PDAC contributes to various aspects of the disease, including enhanced cancer cell growth, alteration of metabolic processes, evasion of the immune system, and development of resistance to therapies [11]. Some other genetic alterations have been identified in pancreatic cancer, including mutations in the tumor suppressor genes *TP53*, *p16*/*CDKN2A* and *SMAD4* [12].

Regarding treatment, patients with operable disease at the time of diagnosis can be treated with a standard therapeutic strategy that involves surgery followed by adjuvant chemotherapy using FOLFIRINOX (fluorouracil, irinotecan, leucovorin, oxaliplatin). This approach is expected to result in a median overall survival of 54.4 months, which is significantly longer compared to the 35 months achieved with single-agent gemcitabine [13]. Patients diagnosed with advanced disease, including locally advanced and metastatic PDAC, can benefit from multiagent chemotherapy regimens such as FOLFIRINOX, gemcitabine/nab-paclitaxel, and nanoliposomal irinotecan/fluorouracil. These treatment approaches have shown a survival advantage of 2 to 6 months when compared to using a single-agent gemcitabine [13]. In patients with germline *BRCA1*/*2* mutations, the poly(adenosine diphosphate-ribose) polymerase inhibitor olaparib, has the potential to improve progression free survival [14].

At the time of diagnosis, pancreas computed tomography (CT) angiography along with chest and pelvis CT scans are used to evaluate the vascular anatomy and disease stage. To obtain a histologic diagnosis and gather material for molecular testing, the recommended procedure is to perform an ultrasound-guided fine-needle core biopsy, which is preferred over fine-needle aspiration [13]. However, the abundance of tumor stroma in the pancreatic tissue affects the negative predictive value of this technique due to sampling error, being sometimes necessary to repeat the procedure in patients with great clinical deterioration [15, 16]. Moreover, these samples are only available at the time of diagnosis, but not during the course of the disease to monitor response to treatment. To date, the carbohydrate antigen 19 − 9 (CA19- 9) is the only blood-based biomarker routinely used to make clinical decisions in pancreatic cancer, with a relatively low sensitivity (79%) and specificity (82%) [17]. There is a relationship between CA19-9 levels and survival in patients with metastatic PDAC [18, 19]. However, in clinical practice there is no consensus on the interpretation of changes in CA19-9 levels throughout the disease [20].

**Fig. 1:**
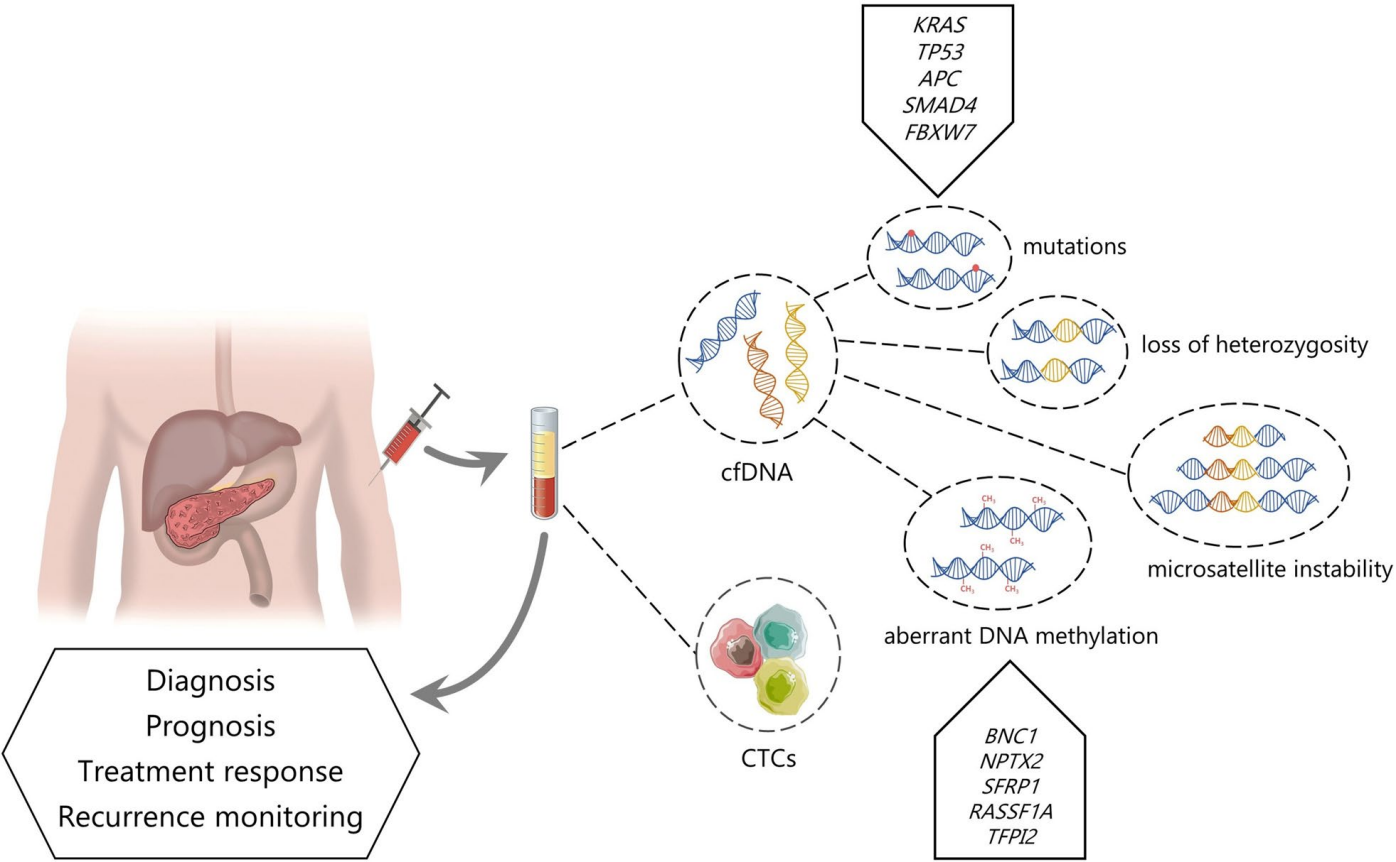
Utility of blood-based liquid biopsy in diagnosis and management of pancreatic cancer. Circulating tumour cells (CTCs) and circulating cell-free DNA (cfDNA), stand as prominent biomarkers in liquid biopsy, providing non-invasive diagnostic, prognostic and therapeutic information. In particular, analysis of cfDNA can reveal tumor-causing genetics alterations, such as mutations, microsatellite instability (MSI), loss-of-heterozygosity (LOH), or aberrant methylation patterns. The mutated genes most frequently detected in cfDNA from pancreatic cancer patients are *KRAS*, *TP53*, *APC*, *SMAD4* and *FBXW7*. Among the circulating biomarkers of methylation in pancreatic cancer are *BNC1*, *NPTX2*, *SFRP1*, *RASSF1A* and *TFPI2*

For all these reasons, the development of alternative blood-based biomarkers for pancreatic cancer is imperative. These biomarkers would aid in early-stage diagnosis, precise patient stratification, selection of appropriate treatments, monitoring of treatment response, evaluating therapy resistance, identification of minimal residual disease and risk of relapse. Liquid biopsy, which refers to the analysis of molecular biomarkers in circulating blood components, has emerged as a promising approach to overcome these challenges in cancer diagnosis and monitoring (Fig. 1). By detecting and analyzing genetic alterations in circulating cell-free DNA (cfDNA), circulating tumor cells (CTCs), and other biomarkers present in the blood, liquid biopsy offers a non-invasive method for assessing tumor characteristics and monitoring treatment response.

Aberrant DNA methylation patterns are frequently observed in cancer cells, and these changes can be reflected in the cfDNA circulating in the blood [21]. The detection of these methylation alterations in the bloodstream holds promise for early cancer detection, potentially leading to improved outcomes through timely intervention. Therefore, this review is aimed to provide an overview of recent advancements in the field of cfDNA methylation analysis in pancreatic cancer. We will first review those studies focusing on cfDNA in pancreatic cancer. Then, we will discuss the relevance of DNA methylation in the context of pancreatic cancer before summarizing recent research in cfDNA methylation, with a particular emphasis on examining its clinical utility. Finally, we will discuss the future directions, clinical translation, and potential integration of cfDNA methylation analysis into routine clinical practice.

The literature search for this review was performed in PubMed in October 2022 with the following MeSH terms and free text used in combination: “Pancreatic cancer” [MeSH], “Pancreatic ductal adenocarcinoma” [MeSH], “cfDNA methylation” [tiab], “circulating free DNA methylation” [tiab], “liquid biopsy” [MeSH], “liquid biopsy” [tiab], and “epigenetics”. Articles for which only the abstract was available or which were not written in English were excluded. Also, the references cited in retrieved articles were examined to identify additional relevant studies.

## Circulating cell-free DNA as blood-based biomarker in pancreatic cancer

Those fragments of DNA that are present in the bloodstream and not contained within cells are referred to as circulating cell-free DNA (cfDNA). This cfDNA originates from various sources, including normal cell turnover, apoptotic or necrotic cells, and tumor cells [22]. Tumor-specific genetic alterations, including mutations, microsatellite instability (MSI), loss of heterozygosity (LOH), and aberrant methylation patterns can be detected in cfDNA [23]. The size of cfDNA varies from 40 to 200 base pairs (bp), with a peak at about 166 bp, although the median overall fragment lengths of cfDNA in healthy individuals have been observed to be larger in comparison to those of cancer patients [24].

Compared to tumor biopsies, cfDNA not only provides a better description of the complete landscape of a tumor but also offers the possibility of repeated sampling and analysis, which allows longitudinal evaluation of dynamic changes in cfDNA concentration, identification of acquired resistance-conferring mutations, and monitoring of clonal evolution [25]. Nevertheless, the analysis of cfDNA as a blood biomarker in cancer also has some limitations. An important aspect in the diagnostic and prognostic utility of cfDNA is its low concentration in plasma, complicating detection and analysis. It has been estimated that there are approximately 10–15 ng of cfDNA per milliliter of plasma and that circulating tumor DNA represents a small fraction of cfDNA in most early-stage cancers [26, 27]. These considerations highlight the requirement for ultra-sensitive detection methods. The most sensitive methods are polymerase chain reaction (PCR)-based methods, such as BEAMing single-molecule PCR [28], TAm-Seq [29], digital PCR [30], and droplet digital PCR [31]. Regarding pancreatic cancer, cfDNA has received increasing attention as a promising biomarker for early detection and prognosis. Thus, recent studies show that genetic alterations in circulating cfDNA in pancreatic cancer are detected in more than 80% of patients with metastatic disease but only in 48% of patients with localized tumors [32].

In pancreatic cancer, liquid biopsy studies performed on microsatellite instability (MSI) are very scarce or non-existent in the case of loss of heterozygosity (LOH). Chakrabarti et al. evaluated whether determination of MSI status in circulating tumor DNA using the Guardant360 technique predicted a robust response to immunotherapy in patients with PDAC. Tissue-based MSI results were concordant with plasma-based G360 results in 83% of patients. Furthermore, in a single patient, MSI was identified in plasma but not in the tumor tissue [33].

The most common blood-based biomarker studies in pancreatic cancer include the analysis of circulating mutations in cfDNA, and the mutated genes most frequently detected are *KRAS*, *TP53*, *APC*, *SMAD4* or *FBXW7* [34]. The concurrence of these mutations with those found in the tumor, strongly emphasizes the potential of cfDNA as a valuable blood-based biomarker for pancreatic cancer. Hence, several studies have confirmed the adverse prognosis associated with the detection of *KRAS* mutations in circulating cfDNA of patients with locally advanced or metastatic PDAC [35–37]. Apart from its prognostic value, some studies have described the detection of *KRAS* mutations in circulating cfDNA as biomarker to monitor treatment response and identify early signs of resistance in pancreatic cancer [38, 39]. However, other authors have not been able to corroborate these findings [40]. In a recent study, the presence of *KRAS* mutations in cfDNA from unresectable pancreatic cancer patients was strongly associated with unfavorable treatment results and suggested this molecular evaluation as biomarker early tumor progression [41].

While the number of studies is currently limited, they highlight the potential of cfDNA as a valuable source of biomarkers for predicting the prognosis of PDAC patients. Furthermore, alterations in cfDNA genetic profiles throughout treatment can serve as early indicators of treatment response or resistance, thereby presenting a promising avenue for utilizing the biomarker’s progression to guide treatment decisions.

Nonetheless, there is a crucial need to advance the development of more sensitive techniques capable of enhance detection tumor-derived circulating DNA, as well as to augment the accuracy of prognosis prediction. The combination of ultrasensitive techniques and the integration of epigenetic markers, such as cfDNA methylation, offer a promising opportunity to increase the sensitivity and specificity of cfDNA analysis in pancreatic cancer (Fig. 1).

## DNA methylation and pancreatic cancer

In recent years, the number of studies attempting to identify methylation markers in pancreatic cancer has increased. Moreover, unlike genetic alterations, DNA methylation is reversible, making it highly valuable from a therapeutic perspective. DNA methylation involves the addition or removal of a methyl group (CH_3_) at the C5 position of cytosine within CpG dinucleotides that are predominantly found in specific genomic regions known as CpG islands. In mammals, the establishment of DNA methylation patterns is primarily mediated by the DNA methyltransferase 3 (DNMT3) family of de novo methyltransferases, including DNMT3A and DNMT3B. Once established, these patterns are subsequently maintained by the action of DNMT1 [42–44]. In normal cells, correct DNA methylation patterns ensure proper and precise regulation of gene expression and maintaining stable gene silencing. Consequently, it is not surprising that the presence of aberrant methylation patterns is widely considered as an epigenetic hallmark in many types of cancer. Notably, both hypo- and hypermethylation events are observed in cancer. Specifically, there is a global decrease of methylated CpG content in gene-poor regions and repetitive sequences. This phenomenon contributes to genomic instability and, although less common, to the activation of previously silenced oncogenes. Conversely, cancer frequently displays localized hypermethylation of gene promoters, resulting in the transcriptional silencing of tumor suppressor genes [45, 46].

There are numerous studies conducted on primary tissue biopsies, cell lines or xenograft models focused on decipher disease-specific methylation patterns in pancreatic diseases to serve as diagnostic or prognostic tool for pancreatic tumor. In pancreatic cancer, almost 80% of cases show upregulation of *DNMT1*, leading to hypermethylation, which is considered the predominant and aberrant epigenetic alteration in pancreatic cancer. The impact of this abnormal hypermethylation is predominantly observed in tumor suppressor genes. The first tumor suppressor gene described in pancreatic cancer as inactivated by aberrant hypermethylation in its promoter was *CDKN2A*/*p16INK4*, which plays a crucial role in inhibiting cell cycle progression the G1 to S phase, ensuring cell cycle arrest [47, 48]. Other studies conducted in fresh frozen tissues of pancreatic exocrine and intraepithelial neoplasms, human pancreatic cancer cell lines, and xenografts, reported hypermethylation and the consequent downregulation in pancreatic cancer of other negative regulators of cell progression through G1 phase, such as the cyclin-dependent kinase inhibitor CDKN1C/p51KIP2 and cyclin CCND2 [49, 50]. Additional tumor suppressor genes with reduced expression due to aberrant hypermethylation in pancreatic tumors are preproenkephalin (*PENK*, hypermethylated in 93.3% of the tumor samples analyzed), the suppressor of cytokine-signaling 1 gene (*SOCS*-1, in 57.1%), protocadherin 10 (PCDH10, in 60.9%), iroquois homeobox 4 (*IRX4*, in 64%), and reprimo (*RPRM*, in 57%), among others [51–55].

In addition to aberrant hypermethylation of tumor suppressor genes in pancreatic cancer, it has been observed that certain genes also undergo aberrant hypomethylation. This hypomethylation predominantly occurs at promoter regions of specific genes, leading to their overexpression, thereby contributing to cell proliferation, survival, and invasiveness in pancreatic cancer cells [56]. One of these genes is the serine protease inhibitor *SERPINB5* (Maspin), that has been reported as completely unmethylated in 87% of pancreatic cancer cell lines (20/23), 94% of xenografts (32/34) and hypomethylated in 86% of primary pancreatic adenocarcinomas (6/7), with an inverse correlation between methylation and mRNA expression level [57]. Indeed, in clinical samples, the presence of unmethylated *SERPINB5* has demonstrated its potential as a specific biomarker for pancreatic tumors, enabling the differentiation of pancreatic ductal adenocarcinoma (PDAC) from pancreatitis [58]. Through multi-omics analysis that combines methylation and expression profiling data, compelling evidence has emerged regarding the upregulation of specific genes in pancreatic cancer tissues associated with their hypomethylation status, including sulfotransferase family 1E member 1 (SULT1E1), insulin-like growth factor 2 mRNA-binding protein 3 (IGF2BP3) and mitogen-activated protein 4 kinase 4 (MAP4K4) [59]. Moreover, the altered methylation and expression profiles of these genes have shown a significant association with overall survival in pancreatic cancer patients, thus suggesting their potential utility as prognostic biomarkers. Several additional genes have shown overexpression as a result of aberrant hypomethylation in pancreatic cancer tissues, including *MUC4* [60], *CLDN4*, *LCN2*, *SFN*, *TFF2*, *S100A4*, *MSLN*, and *PSCA* [56].

In addition, in recent years, 5-hydroxymethylcytosine (5hmC), an oxidized form of 5mC whose biological function is still unclear, has attracted great interest as a potential biomarker for cancer diagnosis and survival. Several studies have reported that 5hmC levels are substantially reduced in human cancers [61–63], and specifically, pancreatic cancer has been described as leading to disease-specific changes in the cell-free hydroxymethylome [64].

In summary, epigenetic alterations play a pivotal role in the initiation and progression of pancreatic cancer. Therefore, the comprehensive analysis of the aberrant epigenetic modifications arising in pancreatic cancer can have significant implications in molecular diagnosis and treatment monitoring.

## Analysis of cfDNA methylation studies in pancreatic cancer

The exploration of methylated biomarkers in plasma for pancreatic cancer is still in its early stages, and the number of studies conducted to date for this purpose is limited.

Analysis of cfDNA methylation patterns in pancreatic cancer has been approached both at the whole genome level and by identifying and describing individual genes or small gene panels. Regarding the whole genome sequencing approach, multiple studies describing signatures have been published [65–67].

In this review, the main interest has been to explore the utility of individual genes or small panels of genes as potential biomarkers with future clinical application (Table 1). One of the first studies addressing the identification of methylated markers for pancreatic cancer in plasma dates from 2007. Jiao et al. examined by methylation specific PCR (MSP) the methylation status of *ppENK* and *p16* genes in plasma samples from 83 patients with untreated pancreatic cancer [48]. Hypermethylation of *ppENK* and *p16* promoters was found in 29.3% and 24.6% of patients, respectively. Moreover, in 9 of 83 patients paired pancreatic tumor tissue sections were available, and hypermethylation of the *p16* and *ppENK* genes was found in 60% and 80%, respectively, of plasma samples from patients whose tumors harbored the same methylation. Authors concluded that plasma DNA may have value as a surrogate for tumor tissues in the detection of epigenetic alterations in pancreatic cancer. However, the obtained sensitivity was too low for a potential diagnostic marker, probably due to the low number of paired plasma/tumor samples that were analyzed. Furthermore, it is important to note that this study lacks data on control groups consisting of both healthy individuals and patients with benign pancreatic diseases.

**Table 1:** Studies on cell-free DNA methylation in plasma/serum as diagnostic and/or prognostic biomarkers for pancreatic adenocarcinoma

Reference	Cases	Controls^a^	Relevant Genes	Method^b^	Results
**Jiao Li et al., 2007 [**48**]**	83 PDAC:	0	*ppENK*	MSP	p16 (9 patients): sensitivity 70%; specificity 100%
	16 I-II				
	37 III		*p16*		
	30 IV				
**Melnikov et al., 2009 [**68**]**	34 PDAC: 19 I-II 2 III 13 IV	30 HC	*CCND2*	MethDet56	Based on the unmethylated status of the composited biomarker: sensitivity 76%; specificity 59%
			*SOCS1*		
			*THBS*		
			*PLAU*		
			*VHL*		
**Ligget et al., 2010 [**69**]**	30 PDAC	30HC 30CP	*BRCA1*^*2*^ *CCND2*^*1,2*^ *CDKN1C*^*1,2*^ *CDKN2B*^*1*^ *DAPK1*^*1*^ *ESR1*^*1*^ *MGMT*^*1*^ *MLH1*^*1,2*^ *MUC2*^*1*^ *MYOD1*^*1*^ *PGK1*^*1*^ *PGR proximal*^*1,2*^ *PGR distal*^*2*^ *RARB*^*1*^ *RB1*^*1*^ *SYK*^*1,2*^	MethDet56	^1^Methylation of 14 gene promoters distinguishes between CP and PDAC: sensitivity 91.2% (95% CI 76.5-97.1); specificity 90.8% (95% CI 76.1-96.8) ^2^Methylation of 8 gene promoters distinguishes between HC and CP: sensitivity 81.7% (95% CI 67.3-90.6); specificity 78% (95% CI 63.8-87.7)
**Melson et al., 2014 [**70**]**	30 PDAC: 18 I-II 5 III 7 IV	30 HC	*VHL*	MethDet56	Combined 5 markers to differentiate PDAC from HC: sensitivity 80%; specificity 66% *GPC3*: sensitivity 63.3%; specificity 83.3%
			*MYF3*		
			*TMS*		
			*GPC3*		
			*SRBC*		
**Park et al., 2012 [**71**]**	16 PDAC: 1 I 8 III 7IV	29 HC	*UCHL1*	MSP + bisulfite sequencing	Higher methylation detection in PDAC compared to HC (p<0.05)
			*NPTX2*		
			*SARP2*		
		13 CP	*ppENK*		Methylated *p16* significantly higher in PDAC than in CP (p = 0.016)
			*p16*		
			*RASSF1A*		
**Park et al., 2012 [**72**]**	104 PDAC:	60 CP	*NPTX2*	qMSP	*NPTX2*: sensitivity 80%; specificity 76%
	24 I-II	5 benign biliary tract stone disease			
	43 III				
	37 IV				
**Kawasaki et al., 2013 [**73**]**	47 PDAC	197: colon, lung, gastric, breast cancers and hepatocarcinoma	*APC*	MSP	Methylation frequencies:
			*DCC*		
			*p16*		*RASSF1A* 34% *APC* 23.4%
			*p14*		*p16* 17% *p14* 14.9%
			*RASSF1A*		*DCC* 6.4%
**Yi et al., 2013 [**74**]**	42 PDAC: 10 I 32 II-IV	26 HC	*BNC1*	MOB	*BNC1*: sensitivity 79% (95% CI 66-91); specificity 89% (95% CI 76-100)
					*ADAMTS1*: sensitivity 48% (95% CI 33-63); specificity 92% (95% CI 82-100)
			*ADAMTS1*		*BNC1*+*ADAMTS1*: sensitivity 81% (95% CI 69-93); specificity 85% (95% CI 71-99)
					90% sensitivity in stage I for both genes
**Henriksen et al., 2016 [**75**]**	95 PDAC: 40 I-II 13 III 42 IV	97 CP	*APC*	MSP + qMSP	Diagnostic prediction model with 8 genes methylation panel that differentiate malign from benign conditions: AUC=0.86 (95% CI 0.81-0.91), sensitivity 76%; specificity 83% Performance of prediction model in early-stages (I-II): AUC=0.86 (95% CI 0.79–0.92), 73% sensitivity; 83% specificity
			*BMP3*		
		59 acute pancreatitis	*BNC1*		
			*MESTv2*		
			*RASSF1A*		
		27 benign conditions	*SFRP1*		
			*SFRP2*		
			*TFPI2*		
**Henriksen et al., 2017 [**76**]**	95 PDAC: 40 I-II 13 III 42 IV	0	*ALX4* ^*1,2*^	MSP	Two methylation-based prognostic prediction models:
			*BNC1* ^*1,2*^		
			*CDKN2B* ^*1,2*^		
			*HIC1* ^*1*^		^1^Methylation of 8 genes that differentiate stage IV from stage I-III disease: AUC=0.87, sensitivity 74%; specificity 87%
			*MLH1* ^*1,2*^		
			*NEUROG1* ^*1,2*^		
			*SEPT9v2* ^*1,2*^		^2^Methylation of 8 genes that differentiate stage I-II from stage III-IV disease: AUC=0.82, sensitivity 73%; specificity 80%
			*SST* ^*1*^		
			*TFPI2* ^*2*^		
			*WNT5A* ^*2*^		
**Henriksen et al., 2017 [**77**]**	95 PDAC: 40 I-II 13 III 42 IV	0	*BNC1*	MSP	Gene hypermethylation based survival prediction model (Hazard ratios (95% CI)): *BNC1* 2.00 (1.26-3.18); *GSTP1* 9.55 (2.70-33.82); *SFRP1* 1.94 (1.24-3.02); *SFRP2* 0.45 (0.27-0.73) and *TFPI2* 2.52 (1.42-4.47)
			*GSTP1*		
			*SFRP1*		
			*SFRP2*		
			*TFPI2*		
**Eissa et al., 2019 [**78**]**	39 PDAC: 37 I-II 2 III-IV	95 HC 8 CP	*BNC1* *ADAMTS1*	qMSP	*BCN1*: AUC=0.79 (95% CI 0.70-0.85), sensitivity 64.1%; specificity 93.7% *ADAMTS1*: AUC=0.91 (95% CI 0.85-0.95) sensitivity 87.2%; specificity 95.8% *BNC1* + *ADAMTS1*: AUC=0.95 (95% CI 0.90-0.98) sensitivity 97.4%; specificity 91.6%
**Li et al., 2019 [**79**]**	57 PDAC	53 HC	*BNC1* *SEPT9*	qMSP	*BCN1*: sensitivity 50.9% (95% CI 37.3-64.4); specificity 88.7% (95% CI 77.0-95.7) *SEPT9*: sensitivity 36.8% (95% CI 24.5-50.7); specificity 96.2% (95% CI 87.0-99.5) *BNC1* + *SEPT9*: sensitivity 64.9% (95% CI 55.0-78.8); specificity 86.8% (95% CI 74.7-94.5) Combined genes + CA19-9: sensitivity 86% (95% CI 74.2-93.7); specificity 81.1% (95% CI 68.0-90.6)
		14 PanIN			
		44 benign conditions			
**Singh et al., 2020 [**80**]**	61 PDAC: 20 I-II 38 III-IV 2 unspecified	22 HC	*UCHL1* *PENK* *NPTX2* *SPARC*	qMSP	Methylation index (MI) of 4 genes higher in PDAC than in HC (p < 0.05) Lower survival in patients with high MI for *SPARC* and *NPTX2* genes (p < 0.05)
		21 CP			
**Shinjo et al., 2020 [**81**]**	47 PDAC: 2 II 41 III-IV 4 unknown	14 HC	*ADAMTS2* *HOXA1* *PCDH10* *SEMA5A* *SPSB4*	MBD-ddPCR	Methylation levels in the 5 genes not significantly different between PDAC and HC 49% of PDAC patients with at least one methylated gene, 49% sensitivity; 86% specificity DNA methylation in ≥ 1 gene and/or *KRAS* mutation: sensitivity 68%; specificity 86%
**Li et al., 2020 [**82**]**	4 PDAC: 2 II 2 III	2 HC	*TRIM73*	MeDIP-seq	Combined 8 gene panel: sensitivity 97.1%; specificity 98%
			*FAM150A*		
			*EPB41L3*		
			*SIX3*		
			*MIR663*		
			*MAPT*		
			*LOC100128977*		
			*LOC100130148*		
**Manoochehri et al., 2020 [**83**]**	30 PDAC: 15 nonmetastatic 15 metastatic	18 HC	*SST*	ddPCR	*SST*: sensitivity 93%; specificity 89%
**Cao et al., 2020 [**84**]**	67 PDAC: 8 I 26 II 17 III 16 IV	97 HC	5mC 5hmC	MeDIP-seq 5hmC sequencing (hMe-Seal)	A 24-feature 5mC model that can discriminate PDAC from HC, sensitivity 82.4%; specificity 100% A 27-feature 5hmC model that can discriminate PDAC from HC, sensitivity 85.7%; specificity 100% The 51-feature model combining 5mC and 5hmC markers: sensitivity 93.8%; specificity 95.5%
**Ying et al., 2021 [**85**]**	22 PDAC:	10 HC	*ADAMTS1* *BNC1* *LRFN5* *PXDN*	MOB-qMSP	Pancreatic cancer detection with 4-gene panel: AUC=0.94, sensitivity 100%; specificity 90%
	3 I				
	15 II				
	1 III-IV				
	3 not available				
**Henriksen et al., 2021 [**86**]**	346 PDAC: 11 I 165 II 33 III 137 IV	25 CP	*APC*	MSP	Validation study of the diagnostic prediction model proposed in Henriksen et al., 2016: AUC=0.77 (95% CI 0.69-0.84) Diagnostic prediction model + CA19-9 in: Resectable disease (I-II): AUC=0.89 (95% CI 0.83-0.95) Unresectable disease (IV): AUC=0.95 (95% CI 0.92-0.98) Entire cohort: AUC=0.85 (95% CI 0.79-0.91)
			*BMP3*		
			*BNC1*		
			*MESTv2*		
			*RASSF1A*		
			*SFRP1*		
			*SFRP2*		
			*TFPI2*		
**Majumder et al., 2021 [**87**]**	170 PDAC: 5 I 45 II 60 III 60 IV	170 HC	*GRIN2D*	TELQAS assay	Methylated DNA marker (MDM) panel: AUC=0.90 (95% CI 0.86-0.94) MDM panel + CA 19-9: AUC=0.97 (95% CI 0.94-0.99), sensitivity 92% (95% CI 83-98); specificity 92% (95% CI 81-100) MDM panel for early-stage detection: AUC=0.84 (95% CI 0.76-0.92) MDM + CA19-9 for early-stage detection: AUC=0.90 (95% CI 0.84-0.97)
			*CD1D*		
			*ZNF781*		
			*FER1L4*		
			*RYR2*		
			*CLEC11A*		
			*AK055957*		
			*LRRC4*		
			*GH05J042948*		
			*HOXA1*		
			*PRKCB*		
			*SHISA9*		
			*NTRK3*		
**Miller et al., 2021 [**88**]**	25 PDAC: 1 I 7 II 4 III 13 IV	20 HC	*ZNF154*	MOB-DREAMing	*ZNF154* for early stage (I-II): AUC=0.87, sensitivity 100% and specificity 80% *ZNF154* for late stage (III-IV): AUC=0.85, sensitivity 94.1% and specificity 80%
**Vrba et al., 2022 [**89**]**	19 PDAC 19 IV	44 benign conditions	*MIR129-2* *LINC01158* *CCDC181* *PRKCB* *TBR1* *ZNF781* *MARCH11* *VWC2* *SLC9A3* *HOXA7*	qMSP	Biomarker set of 10 genes capable of distinguishing malignant from benign cases: AUC=0.999 (95% CI 0.995-1.0) sensitivity 100% and specificity 95% Biomarker set useful for monitoring: methylation decrease after treatment (p=3.9x10^-3^)
**García-Ortiz et al., 2023 [**90**]**	44 PDAC 44 IV	2 HC	*BMP3* *NPTX2* *SFRP1* *SPARC* *TFPI2*	ddPCR	*NPTX2* methylation distinguished between low- and high-risk poor prognosis patients (p-=6.7x10^-3^) *NPTX2* methylation dynamics during patients monitoring predict evolution disease and survival: AUC=0.80 (95% CI 0.66-0.94), sensitivity 85%; specificity 65%

^a^ HC: healthy controls; CP: chronic pancreatitis; PanIN: pancreatic intraepithelial neoplasia

^b^ MSP: Methylation Specific PCR; qMSP: quantitative Methylation Specific PCR; MOB: Methylation On Beads; MBD-ddPCR: Methyl-CpG Binding Domain- digital droplet PCR; MeDIP-seq: Methylated DNA Immunoprecipitation-sequencing; TELQAS: Target Enrichment with Long probe Quantitative Amplified Signal assay; DREAMing: Discrimination of Rare EpiAlleles by Melting

Shortly thereafter another pioneering study was published showing that pancreatic cancer detection could be conducted by using methylation profiling of circulating cfDNA in plasma [68]. Melnikov et al. analyzed the methylation profiles in 30 patients with PDAC and 30 age-matched healthy volunteers. Introducing a novel technique at that time, called MethDet56, the study utilized a microarray test panel comprising 56 frequently methylated genes. This innovative approach aimed to measure the methylation level of target sequences by digesting them with a methylation-sensitive endonuclease and subsequently amplifying the undigested fragments using PCR. A set of five genes (*CCND2*, *PLAU*, *SOCS1*, *THBS*, and *VHL*) was discovered to be hypomethylated in plasma from PDAC patients when compared with healthy controls. This hypomethylation pattern exhibited a sensitivity of 76% and a specificity of 59%. The authors categorized this set of genes as a composite biomarker, establishing its consistent predictive value for the detection of pancreatic cancer using plasma-based methods. Moreover, they argue that the unmethylated status of a promoter is more informative in terms of tumor detection. Nevertheless, it is important to note that these findings have not been validated by subsequent studies, and the use of hypomethylated specific genes in plasma cfDNA as biomarkers for PDAC remains a topic of debate.

The same research group used their developed MethDet56 methodology to compare the methylation of plasma cfDNA in 30 PDAC patients, 30 chronic pancreatitis patients and 30 healthy control individuals, each group with similar age, sex, and ethnic distribution [69]. In an effort to identify specific methylation profiles in plasma, researchers selected the promoters of 8 informative genes (*BRCA1*, *CCND2*, *CDKN1C*, *MLH1*, proximal and distal *PGR* promoter regions, *SYK*, and *VHL*) to distinguish between chronic pancreatitis and healthy controls (78% sensitivity and 81.7% specificity*).* Moreover, they identified fourteen gene promoters (*CCND2*, *CDKN1C*, *CDKN2B*, *DAPK1*, promoter A of *ESR1*, *MGMT*, *MLH1*, *MUC2*, *MYOD1*, *PGK1*, the proximal region of the *PGR* promoter, *RARB*, *RB1*, and *SYK*) as differentially methylated when comparing chronic pancreatitis with PDAC (90.8% sensitivity and 91.2% specificity). Nine of the fourteen gene promoters were specific to chronic pancreatitis, and five were included in both classifiers’ groups. In this shared group of five genes, it was observed that those hypermethylated genes in chronic pancreatitis were hypomethylated in PDAC.

Some years later, the MethDet56 method was applied to investigate whether PDAC and colorectal cancer (CRC) share methylation markers in plasma cfDNA [70]. A seven gene panel (*MDR1*, *SRBC*, *VHL*, *MUC2*, *RB1*, *SYK* and *GPC3*) was identified as the best circulating methylation signature that differentiated either CRC or PDAC from healthy controls. Furthermore, in a more restrictive analysis of this panel, the authors concluded that *GPC3* was the only gene for effectively differentiate between PDAC and healthy controls, whereas *VHL* and *SRBC* were informative genes for both PDAC and CRC.

Park J.W. et al., published two studies in 2012 using MSP technique in plasma for the diagnosis of pancreatic cancer. The first was a pilot study (16 patients with pancreatic cancer, 13 patients with chronic pancreatitis and 29 healthy controls) that used a panel of 6 candidate genes chosen based on the results previously obtained in primary pancreatic cancers and normal pancreatic ductal epithelia by Sato et al., 2003 [71, 91]. Promoters from *UCHL1*, *NPTX2*, *SARP2*, *ppENK*, *p16* and *RASSF1A* genes were found differentially methylated between PDAC patients and healthy controls, but only *p16* promoter was differentially methylated between PDAC patients and those with chronic pancreatitis, a known risk factor for pancreatic cancer. Following these results and previous ones obtained from pancreatic cancer cytology samples [92], these authors focused on methylation status of *NPTX2* in a larger plasma cohort of 104 PDAC patients, 60 chronic pancreatitis patients, and 5 patients with benign biliary tract stone disease [72]. *NPTX2* methylation was significantly higher in the PDAC group (84% of PDAC patients versus 33% and 0% in the chronic pancreatitis and the benign gallstone disease groups respectively; *p = 0.016*), with a sensitivity and specificity of 80% and 76%, respectively, and was positively correlated with worsening tumor stages.

Singh and collaborators analyzed in 2020 three of the six biomarkers examined in 2012 by Park et al. (*UCHL1*, *PENK*, and *NPTX2* gene promoters), adding the *SPARC* gene to the study [80]. Gene Methylation Indices (MI) were calculated from absolute copy numbers obtained by quantitative MSP (qMSP) in cfDNA from 61 PDAC patients, 22 chronic pancreatitis patients, and 21 healthy subjects. The four genes exhibited a significantly higher MI in PDAC than in healthy controls, being *SPARC* MI able to differentiate early stage PDAC from chronic pancreatitis. Moreover, a higher *UCHL1* MI correlated with an advanced stage of the disease; and a higher MI for the *SPARC* and *NPTX2* genes was associated with poor survival in PDAC.

In 2013, Kawasaki et al. studied by using MSP technique the methylation frequency of cell cycle-related genes (*APC*, *DCC*, *p16*, *p14*, and *RASSF1A* ) in cfDNA from patients with different types of cancer, including 47 PDAC patients [73]. The highest methylation frequencies were 34 and 23.4% for *RASSF1A* and *APC* respectively, followed by *p16* and *p14*. However, it should be noted that the study lacked healthy control groups and that the percentage of methylation of *RASSF1A* was similar or even higher in other types of cancer such as hepatocellular carcinoma.

Also in 2013, Yi et al. analyzed by MSP the methylation status of 8 candidate genes selected after cancer-specific methylation filtering of 1,427 unique genes obtained in 4 pancreatic cancer cell lines through transcriptome microarray [74]. *BNC1* and *ADAMTS1* promoter genes showed the highest methylation frequency in primary PDAC tumor samples (91% and 67%, respectively; n = 123) and in premalignant pancreatic intraepithelial neoplasia (PanIN) samples (70% and 25%, respectively; n = 20). These biomarkers were then validated in serum samples (42 PDAC patients and 26 healthy individuals) employing the Methylation On Beads (MOB) method, a nanotechnology that allows capture, retention, and bisulfite treatment of minimal amounts of DNA. Sensitivity reached 79% for *BNC1* and 48% for *ADAMTS1*, which increased to 90% for both genes in stage I PDAC samples. Specificity was 89% for *BNC1* and 92% for *ADAMTS1*. Combining both genes, the sensitivity to detect very early stages of pancreatic cancer was improved (81%), but not the specificity (85%).

This promising panel of biomarkers was validated by qMSP method nine years later by the same group [78] in an independent cohort of 39 PDAC patients, 95 matching age controls and 8 patients with chronic pancreatitis. Methylation of *ADAMTS1* and *BNC1* were detected in 87.2% and 65.1% of PDAC cases versus 4.2% and 6.3% of non-cancer individuals, respectively. The two-gene combination (*ADAMTS1* and/or *BNC1*) improved individual results reaching methylation levels of 97.4% in patients and 8.4% in controls. However, this combined panel also showed methylation in 87.5% of chronic pancreatitis individuals, failing to differentiate PDAC and chronic pancreatitis. According to stage, cfDNA methylation of *ADAMTS1* / *BNC1* combined panel was found in in 100% of patients with stage I, 88.9% of stage IIA, and 100% of stages IIB, III and IV pancreatic cancers, without any improvement when CA19-9 values were incorporated into the analysis. With these results, authors highlight *ADAMTS1* and *BNC1* as robust markers for the early detection of pancreatic cancer in cfDNA during the initial stages of the disease, offering the opportunity of curative tumor resection.

In 2021, seeking to improve the diagnostic potential, the same research group added *LRFN5* and *PXDN* genes to the *ADAMTS1*/*BNC1* combined methylation panel [85]. Methylation levels were measured in 106 FFPE tissue samples (44 PDAC (stage I-IV), 15 PanIN, 24 intraductal papillary mucinous neoplasms, 15 chronic pancreatitis and 8 non-cancerous controls) using MSP and in 32 plasma samples (22 PDAC (stage I-IV) and 10 healthy controls) using MOB followed by qMSP.

The addition of LRFN5/PXDN to the biomarker panel improved the diagnostic accuracy for the detection of premalignant and early-stage cancers, obtaining an AUC of 0.94. Moreover, the sensitivity and specificity obtained in plasma for this 4-gene panel was 100% and 90%, respectively. Of note is the diversity of the population included in the study, which enhances the broad applicability of the findings. However, the plasma sample size was notably smaller than that of tumor tissue. The authors argue that the methylation frequency of their 4-gene panel in cfDNA was comparable, although lower, than in tissue, perhaps due to existing tumor heterogeneity, suggesting that these biomarker genes are critical for tumor clones capable of hematogenous spread.

Henriksen’s group adopted a distinct approach, focusing on the development of predictive models based on cfDNA methylation for use in the diagnosis, survival prediction and prognosis of pancreatic cancer [75–77]. In 2016, Henriksen et al., evaluated a panel of 28 genes selected based on the findings in previous literature, using an optimized bisulfite treatment protocol and two rounds of MSP and qMSP (outer and inner methylation-specific primers and probes) [75]. Cohort were composed of 95 PDAC patients and 3 control groups: (1) 97 chronic pancreatitis patients, (2) 59 acute pancreatitis patients and (3) 27 benign pancreatic conditions patients. Based on multivariable logistic regression analysis, they established a prediction model (age > 65 years) including 8 genes (*APC*, *BMP3*, *BNC1*, *MESTv2*, *RASSF1A*, *SFRP1*, *SFRP2*, and *TFPI2*) able to successfully differentiate malign from benign conditions with a sensitivity of 76% and a specificity of 83%. The authors highlight the independence of this prediction model with the cancer stage, concluding that it could potentially be used as an early blood-based diagnostic tool for pancreatic cancer.

Subsequently, employing the same panel of 28 genes, the same cohort of PDAC patients and the same experimental approaches, these authors only found highly significant differences in the mean number of hypermethylated genes at stage IV, but not at stages I, II or III, suggesting an accumulation of hypermethylated promoter regions during cancer development and progression [76]. They developed prognostic prediction models that were able to distinguish stage IV PDAC patients from those without distant metastasis (stage I, II and III), and patients with potentially resectable PDAC (stage I and II) from those with non-resectable PDAC (stage III and IV).

Also in 2017, this research group leveraged this cohort to establish the correlation between the survival of PDAC patients and hypermethylated genes in plasma-derived cfDNA [77]. They found a significantly lower survival in patients with more than 10 hypermethylated genes in cfDNA, which varied according to pancreatic cancer staging. The final prediction model of survival, developed by multivariable Cox regression analysis, comprised five genes (*BNC1*, *GSTP1*, *SFRP1*, *SFRP2*, and *TFPI2*) in conjunction with an ASA (American Society of Anesthesiologists) physical status score of three, indicating those patients with severe systemic disease. Methylation of all these genes was related with a poor prognosis, except for *SFRP2*, whose methylation was associated with longer survival. It is necessary to emphasize that in the three described studies by Henriksen and colleagues [75–77] hypermethylation was analyzed as a qualitative binary variable, which leads to a loss of quantitative information.

In 2021, an external validation of their previously published diagnostic prediction model (*BMP3*, *RASSF1A*, *BNC1*, *MESTv2*, *TFPI2*, *APC*, *SFRP1* and *SFRP2*) for PDAC [75] was performed by these researchers, also examining the additional effect of CA 19 − 9 serum on the predictive performance of the diagnostic test [86]. Results from MSP of the initial 28-gene panel on cfDNA samples from 346 PDAC (stage I-IV) and 25 chronic pancreatitis patients showed a higher number of hypermethylated genes in PDAC patients compared to chronic pancreatitis patients (8.11 vs. 5.60). Moreover, an AUC of 0.77 was achieved in validation of the diagnostic prediction model, slightly less than in the first study from 2016 (0.86). The authors argue for this difference by explaining that the primary study was based on training data, which likely produced an overestimation of test performance due to overfitting. Combining this test with serum CA19-9 values, an AUC of 0.85 (0.93 in the primary study) was achieved, allowing the authors to point out that the joint use of both markers could serve as a clinically useful diagnostic tool for PDAC. It is important to consider that, in addition to the low number of control individuals included in this validation analysis, cases and controls were not matched for age or smoking, both of which are important factors that can affect methylation status.

The *BNC1* and *SEPT9* genes, previously described as potential circulating biomarkers in pancreatic cancer [74, 76] were re-analyzed in 2019 by Xiao-Bin Li et al. [79]. Significant differences were found in the circulating methylation levels of both genes by qMSP in 57 PDAC patients, 14 patients with PanIN lesions, 44 with benign conditions and 53 healthy controls, with higher levels in the group of patients with tumors. The sensitivity and specificity of these markers used in conjunction with CA19-9 for the diagnosis of pancreatic cancer was 86% and 81.1%, respectively. However, it is necessary to point out that these markers may be methylated in around one third of benign pancreatic diseases, and in other cancers, such as colorectal cancer, lung cancer and hepatocellular carcinoma [93–95].

In 2020, Shinjo and colleagues pointed to *ADAMTS2*, *HOXA1*, *PCDH10*, *SEMA5A* and *SPSB4* as the most highly/frequently methylated genes in pancreatic cancer tissues with *KRAS* mutations after performing an Illumina Infinium genome-wide DNA methylation analysis, achieving a sensitivity of 98%. Subsequent validation of the methylation status of these five marker genes in serum samples (47 PDAC patients and 14 normal controls) was carried out using a novel and sensitive method consisting of the enrichment of the coupled methyl-CpG binding protein with a digital PCR method (MBD-ddPCR) [81]. Although no significant differences were observed between cancer patients and controls, 49% of PDAC patients had at least one gene methylated, leading to a 49% sensitivity and an 86% specificity. The combination of the cfDNA methylation status and the *KRAS* mutation improved the diagnostic performance, reaching a sensitivity of 68% and a specificity of 86%. A question that remains unanswered is how cfDNA methylation patterns would compare if a group of patients with benign pancreatic disease were included in the study.

Still in 2020, Li and colleagues identified 143 hypermethylated differentially methylated regions (DMRs) derived from 70 genes in cfDNA from 4 PDAC patients and 2 healthy controls using MeDIP-seq technology, that combines immunoprecipitation with anti-5-methylcytosine antibodies and DNA sequencing [82]. The 143 candidate DMRs were further analyzed using genomic data repositories (TCGA and GEO) by the Least Absolute Shrinkage and Selection Operator (LASSO) method, being able to select eight markers (*TRIM73*, *FAM150A*, *EPB41L3*, *SIX3*, *MIR663*, *MAPT*, *LOC100128977* and *LOC100130148*) that significantly distinguished PDAC patients from healthy individuals with a sensitivity of 97.1% and a specificity of 98.0%. Finally, the results of the Kaplan-Meier survival analysis concluded that these eight markers may serve as potential biomarkers for early diagnosis of pancreatic cancer, but not for prognosis.

Manoochehri and collaborators described in 2020 *SST* gene hypermethylation and downregulation of *SST* gene across various tumor types, including pancreatic cancer, performing as a pan-cancer molecular biomarker [83]. Combining selected DMRs from a genome-wide DNA methylation analysis performed on different tissue samples with available expression profiling data from previous studies results in *SST* being the only candidate gene involved in cell proliferation, invasion, migration, cell death and apoptosis, as well as in gastrointestinal function. Verification and validation of hypermethylation and downregulation of the *SST* gene in PDAC tissue samples, via bisulfite restriction, pyrosequencing, qPCR, and analysis of data available in the TCGA and GEO repositories, proved that *SST* hypermethylation and expression have prognostic value and are associated with the survival rate of PDAC patients. Moreover, in agree with Henriksen et al. [75], results from digital droplet PCR (ddPCR) methylation analysis of *SST* allele in 30 plasma samples from PDAC patients and 18 healthy controls revealed a high diagnostic sensitivity (93%) and specificity (89%). Despite these outcomes, *SST* methylation cannot be used as specific marker for pancreatic cancer, and *SST* hypermethylation most likely has the potential as a blood-based pan-cancer biomarker for a wide range of tumors for initial stratification into high and low risk groups.

Feng Cao et al. carried out in 2020 both 5-hydroxymethylcytosine (5hmC) sequencing and cfMeDIP-seq to develop a robust and non-invasive approach using 5-methylcytosine (5mC) and 5hmC markers from cfDNA for the detection of PDAC [84]. By comparing the distributions of the 5mC and 5hmC peaks, they selected a set of 24 and 27 5mC and 5hmC profiles, respectively, that distinguish with a high precision between PDAC and healthy groups, in both a training and a validation set. Furthermore, the integrated prediction model combining 5hmC and 5mC features demonstrated higher prediction sensitivity, particularly in early-stage PDAC samples (87.5% sensitivity in the integrated, 75% and 62.5% in the 5mC and 5hmC models, respectively), supporting the possibility of applying the combined circulating cell-free 5mC and 5hmC biomarkers for a more accurate cancer diagnosis.

Majumder et al. set out to conduct in 2021 a plasma performance assay for 13 methylated DNA markers previously identified by them via RRBS (Reduced Representation Bisulfite Sequencing) libraries in tissues from patients with PDAC [87, 96]. Plasma samples from two independent cohorts of PDAC cases at all stages (170) and cancer-free control subjects (170) were analyzed using the TELQAS assay (Target Enrichment with Long probe Quantitative Amplified Signal) for *GRIN2D*, *CD1D*, *CLEC11A*, *AK055957*, *ZNF781*, *PRKCB*, *FER1L4*, *HOXA1*, *RYR2*, *LRRC4*, *GH05J042948*, *SHISA9* and *NTRK3*. The proposed panel allowed detection in 79%, 82%, 94% and 99% of the cases of stages I, II, III and IV respectively, and reached specificity and sensitivity values of up to 94% and 82% when the markers were combined with CA19-9. This work represents the largest study reporting results of a diagnostic methylation biomarker panel in PDAC patients. However, a cohort of patients with chronic pancreatitis should have been included to reinforce the potential of the described panel.

Based on a proof-of-concept study, Miller et al. targeted *ZNF154* methylation as a suitable biomarker for blood screening of multiple cancers, including pancreatic cancer [88]. They analyzed *ZNF154* methylation at a specific CG position with Illumina 450 K methylation TCGA data derived from PDAC and cancer-free donors tissue samples. Additionally, they collected mutation data from these same samples using cBioPortal. *ZNF4* was found to be hypermethylated in 86.7% of PDAC samples versus 95.3% mutated in the common set of PDAC cancer genes (90.7% in *KRAS* and 4.6% in *TP53*, *SMAD4* or *CDKN2A*). Next, they examined the methylation status of 14 *ZNF154* CpG sites (including the Illumina CG position) in plasma samples by combining MOB and a PCR-based high resolution DNA melting approach (DREAMing). Considering that the study cohort was small (8 stages I-II, 17 stages III-IV and 20 normal controls), a sensitivity of 94.1% and a specificity of 80% were obtained for late-stage pancreas (AUC = 0.85), reaching values of 100% and 80% respectively for early-stage pancreas (AUC = 0.87). Furthermore, they observed: (I) no detectable *KRAS* mutant cfDNA in early-stage samples; (II) statistically significant higher median *KRAS* mutant allele frequencies in late-stage PDAC cases compared with controls; and (III) an AUC of 0.67. Authors conclude that *ZNF154* shows promising potential as a liquid biopsy-based laboratory testing for cancer screening. propose future validations using a larger set of plasma samples encompassing different cancer types and stages, and suggest considering the inclusion of this marker in a clinical trial.

Finally, the use of cfDNA methylation for monitoring disease progression and response to treatment is an important aspect that has been rarely addressed in pancreatic cancer. In this regard, Vrba and colleagues tested the ability of a novel ten-genes DNA methylation signature to evaluate tumor response by analizing pairs of blood samples taken before and 4 weeks after treatment from 9 metastatic PDAC patients using quantitative MSP. Although the cohort and monitoring time were limited, their results showed a statistically significant decrease in the biomarker signal in all treated patients [89].

In a recent study by our group, *NPTX2* methylation levels was analyzed in plasma from 44 metastatic PDAC patients using ddPCR to evaluate its utility for prognosis and monitoring disease progression. Significantly, we demonstrated that the circulating *NPTX2* methylation levels not only serve as a valuable prognostic biomarker but also offer a practical tool for monitoring metastatic PDAC patients [90]. Thus, correlations were observed between changes in *NPTX2* methylation levels and disease progression as well as response to therapy, surpassing CA19-9 in predicting disease evolution in mPDAC patients. Moreover, in many cases, an elevation in circulating *NPTX2* methylation levels preceded the detection of disease progression by CT imaging [90].

## Challenges and conclusions

Circulating methylated DNA hold promise as noninvasive biomarker for pancreatic cancer detection and management. In this review, we have updated research progress on DNA methylation in liquid biopsies as diagnostic or prognostic tools for PDAC .

A persistent and pressing challenge within the field of liquid biopsy, especially when dealing with cfDNA, lies in the imperative need for standardization and clinical validation of techniques. This is essential to enable the transition from fundamental research to the ambit of clinical trials and ultimately advance the field. Both the methodological procedures and the specific targets for analysis are yet to be fully standardized. Thus, to achieve harmonization across laboratories and establish one or more genes as clinically applicable cfDNA-based epigenetic biomarkers for pancreatic cancer, it its crucial to validate them in substantial cohorts of patients and healthy individuals. Furthermore, unification of identification and detection techniques is neccesary to facilitate result comparison between studies, thereby also also ensuring the clinical feasability of the developed methods.

On the other hand, it must be emphasized that epigenetic alterations detected by using a single biomarker are not able to capture the complex biology of the disease. In this sense, the combination of multiple biomarkers can undoubtedly boost the predictive power and allow early diagnosis, prediction of prognosis and treatment response.

**Fig. 2:**
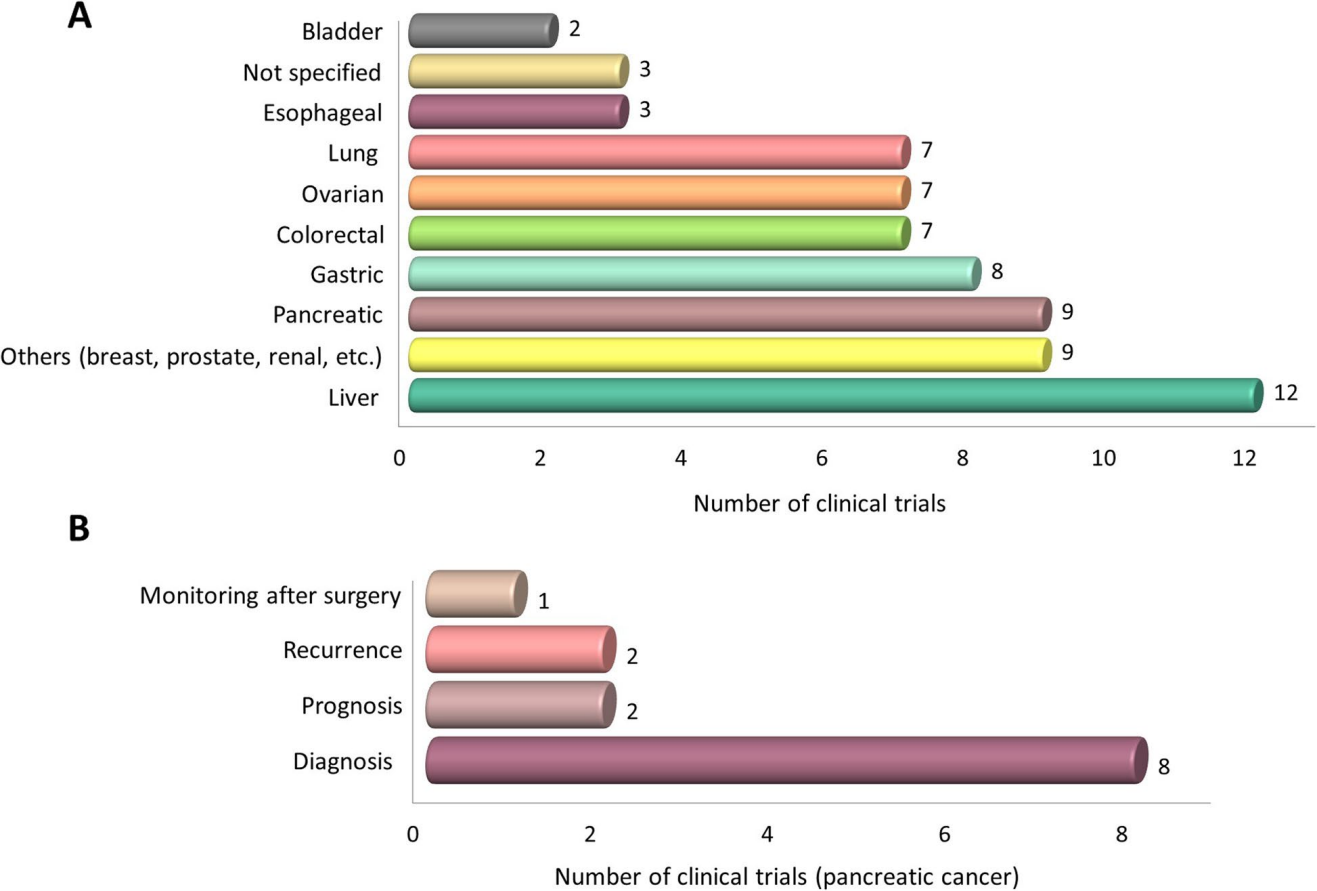
Graphical landscape of current clinical trials using cfDNA methylation in cancer and goals of clinical intervention in cfDNA methylation-based pancreatic cancer trials. **A** Data refer to a total of 67 studies for different cancer types within 47 clinical trials. **B** Data refer to a total of 13 studies with different endpoints within 9 pancreatic cancer clinical trials. Information obtained from https://clinicaltrials.gov/ in June 2023, using the following search terms: [condition or disease: “cancer”]; [other terms: “cfDNA methylation”]; [study status: “recruiting and completed”]

Currently, there are 47 clinical trials investigating the diagnostic and prognostic utility of cfDNA methylation markers in cancer (https://clinicaltrials.gov/ct2/home). Interestingly, nine of them are focused on the validation of methylation blood-circulating biomarkers in pancreatic cancer, and early diagnosis of the disease is the main aim in the majority (70%) of these studies (Fig. 2), although no specific information on which genes are analyzed is provided.

In conclusion, the incorporation of circulating cell-free DNA methylation in clinical and precision medicine for pancreatic cancer is a promising reality on the horizon. For this, it is essential to join efforts to corroborate its efficacy and utility with more well-designed studies that incorporate more sensitive or innovative techniques, as well as an increased number of clinical trials on a large-scale population scale. Finally, collaborative research and shared resources can also pave the way for the incorporation of this innovative approach in clinical practice.

## Data Availability

Not applicable.

## Abbreviations

CT: Computed tomography
cfDNA: Cell-free DNA
CTCs: Circulating tumour cells
ddPCR: Digital droplet PCR
MAF: Mutant allele fraction
MOB: Methylation On Beads
MSP: Methylation Specific PCR
PDAC: Pancreatic ductal adenocarcinoma

## References

[1] Bray F, Ferlay J, Soerjomataram I, Siegel RL, Torre LA, Jemal A (2018). Global cancer statistics 2018: GLOBOCAN estimates of incidence and mortality worldwide for 36 cancers in 185 countries. Cancer J Clin..

[2] Siegel RL, Miller KD, Jemal A (2019). Cancer statistics, 2019. CA Cancer J Clin.

[3] Ferlay J, Colombet M, Soerjomataram I, Dyba T, Randi G, Bettio M, Gavin A, Visser O, Bray F (2018). Cancer incidence and mortality patterns in Europe: estimates for 40 countries and 25 major cancers in 2018. Eur J Cancer.

[4] Partyka O, Pajewska M, Kwasniewska D (2023). Overview of pancreatic Cancer epidemiology in Europe and Recommendations for Screening in High-Risk populations. Cancers (Basel).

[5] Wong W, Raufi AG, Safyan RA, Bates SE, Manji GA (2020). BRCA mutations in Pancreas Cancer: Spectrum, Current Management, Challenges and Future prospects. Cancer Manag Res.

[6] Klein AP (2021). Pancreatic cancer epidemiology: understanding the role of lifestyle and inherited risk factors. Nat Rev Gastroenterol Hepatol.

[7] Lambert A, Schwarz L, Borbath I, Henry A, Van Laethem JL, Malka D, Ducreux M, Conroy T (2019). An update on treatment options for pancreatic adenocarcinoma. Ther Adv Med Oncol.

[8] Carrato A, Falcone A, Ducreux M (2015). A systematic review of the Burden of Pancreatic Cancer in Europe: real-world impact on Survival, Quality of Life and costs. J Gastrointest Cancer.

[9] Walter FM, Mills K, Mendonça SC (2016). Symptoms and patient factors associated with diagnostic intervals for pancreatic cancer (SYMPTOM pancreatic study): a prospective cohort study. Lancet Gastroenterol Hepatol.

[10] Bryant KL, Mancias JD, Kimmelman AC, Der CJ (2014). KRAS: feeding pancreatic cancer proliferation. Trends Biochem Sci.

[11] Zhang Z, Zhang H, Liao X, Tsai HI (2023). KRAS mutation: the booster of pancreatic ductal adenocarcinoma transformation and progression. Front Cell Dev Biol.

[12] Saiki Y, Horii A (2014). Molecular pathology of pancreatic cancer. Pathol Int.

[13] Park W, Chawla A, O’Reilly EM (2021). Pancreatic Cancer: a review. JAMA.

[14] Golan T, Hammel P, Reni M (2019). Maintenance olaparib for germline BRCA-Mutated metastatic pancreatic Cancer. N Engl J Med.

[15] DiPardo BJ, Winograd P, Court CM, Tomlinson JS (2018). Pancreatic cancer circulating tumor cells: applications for personalized oncology. Expert Rev Mol Diagn.

[16] Baek HW, Park MJ, Rhee YY, Lee KB, Kim MA, Park IA (2015). Diagnostic accuracy of endoscopic ultrasound-guided fine needle aspiration cytology of pancreatic lesions. J Pathol Transl Med.

[17] Huang Z, Liu F (2014). Diagnostic value of serum carbohydrate antigen 19 – 9 in pancreatic cancer: a meta-analysis. Tumour Biol.

[18] Saad ED, Machado MC, Wajsbrot D, Abramoff R, Hoff PM, Tabacof J, Katz A, Simon SD (2002). Pretreatment CA 19 – 9 level as a prognostic factor in patients with advanced pancreatic cancer treated with gemcitabine. Int J Gastrointest Cancer.

[19] Maisey NR, Norman AR, Hill A, Massey A, Oates J, Cunningham D (2005). CA19-9 as a prognostic factor in inoperable pancreatic cancer: the implication for clinical trials. Br J Cancer.

[20] Bauer TM, El-Rayes BF, Li X, Hammad N, Philip PA, Shields AF, Zalupski MM, Bekaii-Saab T (2013). Carbohydrate antigen 19 – 9 is a prognostic and predictive biomarker in patients with advanced pancreatic cancer who receive gemcitabine-containing chemotherapy: a pooled analysis of 6 prospective trials. Cancer.

[21] Dor Y, Cedar H (2018). Principles of DNA methylation and their implications for biology and medicine. Lancet.

[22] Ranucci R (2019). Cell-free DNA: applications in different Diseases. Methods Mol Biol.

[23] Lianidou E, Hoon D (2018). Circulating tumor cells and circulating tumor DNA. Principles Applications Mol Diag..

[24] Cristiano S, Leal A, Phallen J (2019). Genome-wide cell-free DNA fragmentation in patients with cancer. Nature.

[25] Bronkhorst AJ, Ungerer V, Holdenrieder S (2019). The emerging role of cell-free DNA as a molecular marker for cancer management. Biomol Detect Quantif.

[26] Jaworski JJ, Morgan RD, Sivakumar S (2020). Circulating cell-free Tumour DNA for early detection of pancreatic Cancer. Cancers (Basel).

[27] Diehl F, Schmidt K, Choti MA (2008). Circulating mutant DNA to assess tumor dynamics. Nat Med.

[28] Diehl F, Li M, He Y, Kinzler KW, Vogelstein B, Dressman D (2006). BEAMing: single-molecule PCR on microparticles in water-in-oil emulsions. Nat Methods.

[29] Forshew T, Murtaza M, Parkinson C (2012). Noninvasive identification and monitoring of cancer mutations by targeted deep sequencing of plasma DNA. Sci Transl Med.

[30] Taly V, Pekin D, Benhaim L (2013). Multiplex picodroplet digital PCR to detect KRAS mutations in circulating DNA from the plasma of colorectal cancer patients. Clin Chem.

[31] Hindson BJ, Ness KD, Masquelier DA (2011). High-throughput Droplet Digital PCR system for Absolute quantitation of DNA Copy Number. Anal Chem.

[32] Bettegowda C, Sausen M, Leary RJ (2014). Detection of circulating tumor DNA in early- and late-stage human malignancies. Sci Transl Med.

[33] Chakrabarti S, Bucheit L, Starr JS (2022). Detection of microsatellite instability-high (MSI-H) by liquid biopsy predicts robust and durable response to immunotherapy in patients with pancreatic cancer. J Immunother Cancer.

[34] Zill OA, Greene C, Sebisanovic D (2015). Cell-free DNA Next-Generation sequencing in Pancreatobiliary Carcinomas. Cancer Discov.

[35] Kim MK, Woo SM, Park B (2018). Prognostic implications of Multiplex Detection of KRAS mutations in cell-free DNA from patients with pancreatic ductal adenocarcinoma. Clin Chem.

[36] Mohan S, Ayub M, Rothwell DG (2019). Analysis of circulating cell-free DNA identifies KRAS copy number gain and mutation as a novel prognostic marker in pancreatic cancer. Sci Rep.

[37] Toledano-Fonseca M, Cano MT, Inga E (2020). Circulating cell-free DNA-Based liquid biopsy markers for the non-invasive prognosis and monitoring of metastatic pancreatic Cancer. Cancers (Basel).

[38] Kruger S, Heinemann V, Ross C (2018). Repeated mutKRAS ctDNA measurements represent a novel and promising tool for early response prediction and therapy monitoring in advanced pancreatic cancer. Ann Oncol.

[39] Sugimori M, Sugimori K, Tsuchiya H (2020). Quantitative monitoring of circulating tumor DNA in patients with advanced pancreatic cancer undergoing chemotherapy. Cancer Sci.

[40] Bernard V, Kim DU, San Lucas FA (2019). Circulating nucleic acids are Associated with Outcomes of patients with pancreatic Cancer. Gastroenterology.

[41] Watanabe F, Suzuki K, Aizawa H, Endo Y, Takayama Y, Kakizawa N, Kato T, Noda H, Rikiyama T (2023). Circulating tumor DNA in molecular assessment feasibly predicts early progression of pancreatic cancer that cannot be identified via initial imaging. Sci Rep.

[42] Okano M, Xie S, Li E (1998). Cloning and characterization of a family of novel mammalian DNA (cytosine-5) methyltransferases. Nat Genet.

[43] Yen RW, Vertino PM, Nelkin BD (1992). Isolation and characterization of the cDNA encoding human DNA methyltransferase. Nucleic Acids Res.

[44] Cheng X, Blumenthal RM (2008). Mammalian DNA methyltransferases: a structural perspective. Structure.

[45] Kulis M, Esteller M (2010). DNA methylation and cancer. Adv Genet.

[46] Baylin SB, Jones PA (2016). Epigenetic determinants of Cancer. Cold Spring Harb Perspect Biol.

[47] Schutte M, Hruban RH, Geradts J (1997). Abrogation of the Rb/p16 tumor-suppressive pathway in virtually all pancreatic carcinomas. Cancer Res.

[48] Jiao Li JZ, Manal M, Hassan DB, Evans JL, Abbruzzese, Li D. Kras mutation and p16 and ppENK promoter hypermetilation in plasma DNA of pancreatic cancer patients: in relation to cigarette smoking. 2007;34(1):55–62. 10.1097/01.mpa.0000246665.68869.d4 PMC1905887 17198183

[49] Matsubayashi H, Sato N, Fukushima N, Yeo CJ, Walter KM, Brune K, Sahin F, Hruban RH, Goggins M (2003). Methylation of cyclin D2 is observed frequently in pancreatic cancer but is also an age-related phenomenon in gastrointestinal tissues. Clin Cancer Res.

[50] Sato N, Matsubayashi H, Abe T, Fukushima N, Goggins M (2005). Epigenetic down-regulation of CDKN1C/p57KIP2 in pancreatic ductal neoplasms identified by gene expression profiling. Clin Cancer Res.

[51] Fukushima N, Sato N, Ueki T, Rosty C, Walter KM, Wilentz RE, Yeo CJ, Hruban RH, Goggins M (2002). Aberrant methylation of preproenkephalin and p16 genes in pancreatic intraepithelial neoplasia and pancreatic ductal adenocarcinoma. Am J Pathol.

[52] Komazaki T, Nagai H, Emi M (2004). Hypermethylation-associated inactivation of the SOCS-1 gene, a JAK/STAT inhibitor, in human pancreatic cancers. Jpn J Clin Oncol.

[53] Curia MC, Fantini F, Lattanzio R, Tavano F, Di Mola F, Piantelli M, Battista P, Di Sebastiano P, Cama A (2019). High methylation levels of PCDH10 predict poor prognosis in patients with pancreatic ductal adenocarcinoma. BMC Cancer.

[54] Chakma K, Gu Z, Abudurexiti Y, Hata T, Motoi F, Unno M, Horii A, Fukushige S (2020). Epigenetic inactivation of IRX4 is responsible for acceleration of cell growth in human pancreatic cancer. Cancer Sci.

[55] Sato N, Fukushima N, Matsubayashi H, Iacobuzio-Donahue CA, Yeo CJ, Goggins M (2006). Aberrant methylation of Reprimo correlates with genetic instability and predicts poor prognosis in pancreatic ductal adenocarcinoma. Cancer.

[56] Sato N, Maitra A, Fukushima N, van Heek NT, Matsubayashi H, Iacobuzio-Donahue CA, Rosty C, Goggins M (2003). Frequent hypomethylation of multiple genes overexpressed in pancreatic ductal adenocarcinoma. Cancer Res.

[57] Sato N, Fukushima N, Matsubayashi H, Goggins M (2004). Identification of maspin and S100P as novel hypomethylation targets in pancreatic cancer using global gene expression profiling. Oncogene.

[58] Mardin WA, Ntalos D, Mees ST, Spieker T, Senninger N, Haier J, Dhayat SA (2016). SERPINB5 promoter hypomethylation differentiates pancreatic ductal Adenocarcinoma from Pancreatitis. Pancreas.

[59] Chen H, Kong Y, Yao Q, Zhang X, Fu Y, Li J, Liu C, Wang Z (2019). Three hypomethylated genes were associated with poor overall survival in pancreatic cancer patients. Aging.

[60] Zhu Y, Zhang JJ, Zhu R, Zhu Y, Liang WB, Gao WT, Yu JB, Xu ZK, Miao Y (2011). The increase in the expression and hypomethylation of MUC4 gene with the progression of pancreatic ductal adenocarcinoma. Med Oncol.

[61] Jin SG, Jiang Y, Qiu R, Rauch TA, Wang Y, Schackert G, Krex D, Lu Q, Pfeifer GP (2011). 5-Hydroxymethylcytosine is strongly depleted in human cancers but its levels do not correlate with IDH1 mutations. Cancer Res.

[62] Kudo Y, Tateishi K, Yamamoto K (2012). Loss of 5-hydroxymethylcytosine is accompanied with malignant cellular transformation. Cancer Sci.

[63] Yang H, Liu Y, Bai F (2013). Tumor development is associated with decrease of TET gene expression and 5-methylcytosine hydroxylation. Oncogene.

[64] Song CX, Yin S, Ma L (2017). 5-Hydroxymethylcytosine signatures in cell-free DNA provide information about tumor types and stages. Cell Res.

[65] Liu Z, Wang Z, Jia E, Ouyang T, Pan M, Lu J, Ge Q, Bai Y (2019). Analysis of genome-wide in cell free DNA methylation: progress and prospect. Analyst.

[66] Liu MC, Oxnard GR, Klein EA, Swanton C, Seiden MV, Consortium C (2020). Sensitive and specific multi-cancer detection and localization using methylation signatures in cell-free DNA. Ann Oncol.

[67] Klein EA, Richards D, Cohn A (2021). Clinical validation of a targeted methylation-based multi-cancer early detection test using an independent validation set. Ann Oncol.

[68] Melnikov AA, Scholtens D, Talamonti MS, Bentrem DJ, Levenson VV (2009). Methylation profile of circulating plasma DNA in patients with pancreatic cancer. J Surg Oncol.

[69] Liggett T, Melnikov A, Yi QL, Replogle C, Brand R, Kaul K, Talamonti M, Abrams RA, Levenson V (2010). Differential methylation of cell-free circulating DNA among patients with pancreatic cancer versus chronic pancreatitis. Cancer.

[70] Melson J, Li Y, Cassinotti E (2014). Commonality and differences of methylation signatures in the plasma of patients with pancreatic cancer and colorectal cancer. Int J Cancer.

[71] Park JWBI, Kim YT (2012). Preliminary Study analyzing the methylated genes in the plasma of patients with pancreatic Cancer. Scand J Surg.

[72] Park JKJR, Yoon WJ, Lee SH, Lee GY, Jeong KS, Jeong K-S, Kim Y-T (2012). Yong Bum Yoon: the role of quantitative NPTX2 hypermethylation as a novel serum diagnostic marker in pancreatic Cancer. Pancreas.

[73] Kawasaki H, Igawa E, Kohosozawa R, Kobayashi M, Nishiko R, Abe H (2013). Detection of aberrant methylation of tumor suppressor genes in plasma from cancer patients. Personalized Med Universe.

[74] Yi JMaG AA, Bailey VJ, Downing SR (2013). Novel methylation biomarker panel for the early detection of pancreatic cancer. Clin Cancer Res.

[75] Henriksen SD, Madsen PH, Larsen AC, Johansen MB, Drewes AM, Pedersen IS, Krarup H, Thorlacius-Ussing O (2016). Cell-free DNA promoter hypermethylation in plasma as a diagnostic marker for pancreatic adenocarcinoma. Clin Epigenetics.

[76] Henriksen SD, Madsen PH, Larsen AC, Johansen MB, Pedersen IS, Krarup H, Thorlacius-Ussing O (2017). Promoter hypermethylation in plasma-derived cell-free DNA as a prognostic marker for pancreatic adenocarcinoma staging. Int J Cancer.

[77] Henriksen SD, Madsen PH, Larsen AC, Johansen MB, Pedersen IS, Krarup H, Thorlacius-Ussing O (2017). Cell-free DNA promoter hypermethylation in plasma as a predictive marker for survival of patients with pancreatic adenocarcinoma. Oncotarget.

[78] Eissa MA, Lerner L, Abdelfatah E (2019). Promoter methylation of ADAMTS1 and BNC1 as potential biomarkers for early detection of pancreatic cancer in blood. Clin Epigenetics.

[79] Li XB, Ma J, Liu ZW (2019). Non-invasive detection of pancreatic cancer by measuring DNA methylation of basonuclin 1 and Septin 9 in plasma. Chin Med J (Engl).

[80] Singh N, Rashid S, Rashid S, Dash NR, Gupta S, Saraya A (2020). Clinical significance of promoter methylation status of tumor suppressor genes in circulating DNA of pancreatic cancer patients. J Cancer Res Clin Oncol.

[81] Shinjo K, Hara K, Nagae G (2020). A novel sensitive detection method for DNA methylation in circulating free DNA of pancreatic cancer. PLoS ONE.

[82] Li S, Wang L, Zhao Q, Wang Z, Lu S, Kang Y, Jin G, Tian J (2020). Genome-wide analysis of cell-free DNA methylation profiling for the early diagnosis of pancreatic Cancer. Front Genet.

[83] Manoochehri M, Wu Y, Giese NA (2020). SST gene hypermethylation acts as a pan-cancer marker for pancreatic ductal adenocarcinoma and multiple other tumors: toward its use for blood-based diagnosis. Mol Oncol.

[84] Cao F, Wei A, Hu X (2020). Integrated epigenetic biomarkers in circulating cell-free DNA as a robust classifier for pancreatic cancer. Clin Epigenetics.

[85] Ying L, Sharma A, Chhoda A (2021). Methylation-based cell-free DNA signature for early detection of pancreatic Cancer. Pancreas.

[86] Henriksen SD, Stubbe BE, Madsen PH (2021). Cell-free DNA promoter hypermethylation as a diagnostic marker for pancreatic ductal adenocarcinoma - an external validation study. Pancreatology.

[87] Majumder S, Taylor WR, Foote PH (2021). High detection rates of pancreatic Cancer across stages by plasma assay of Novel Methylated DNA markers and CA19-9. Clin Cancer Res.

[88] Miller BF, Petrykowska HM, Elnitski L (2021). Assessing ZNF154 methylation in patient plasma as a multicancer marker in liquid biopsies from colon, liver, ovarian and pancreatic cancer patients. Sci Rep.

[89] Vrba L, Futscher BW, Oshiro M (2022). Liquid biopsy, using a novel DNA methylation signature, distinguishes pancreatic adenocarcinoma from benign pancreatic disease. Clin Epigenetics.

[90] Garcia-Ortiz MV, Cano-Ramirez P, Toledano-Fonseca M (2023). Circulating NPTX2 methylation as a non-invasive biomarker for prognosis and monitoring of metastatic pancreatic cancer. Clin Epigenetics.

[91] Sato F (2003). Discovery of Novel Targets for Aberrant Methylation in Pancreatic Carcinoma..

[92] Park JK, Ryu JK, Lee KH (2007). Quantitative analysis of NPTX2 hypermethylation is a promising molecular diagnostic marker for pancreatic cancer. Pancreas.

[93] Shames DS, Girard L, Gao B (2006). A genome-wide screen for promoter methylation in lung cancer identifies novel methylation markers for multiple malignancies. PLoS Med.

[94] Song L, Peng X, Li Y, Xiao W, Jia J, Dong C, Gong Y, Zhou G, Han X (2017). The SEPT9 gene methylation assay is capable of detecting colorectal adenoma in opportunistic screening. Epigenomics.

[95] Oussalah A, Rischer S, Bensenane M (2018). Plasma mSEPT9: a novel circulating cell-free DNA-Based epigenetic biomarker to diagnose Hepatocellular Carcinoma. EBioMedicine.

[96] Kisiel JB, Raimondo M, Taylor WR (2015). New DNA methylation markers for pancreatic Cancer: Discovery, tissue validation, and Pilot Testing in Pancreatic Juice. Clin Cancer Res.

